# Trivalent AMH-INH-RFRP DNA Vaccine Enhances Estrus and Ovulation Rates in Buffaloes

**DOI:** 10.3390/ani16142249

**Published:** 2026-07-21

**Authors:** Chao Chen, Xinxin Zhang, Pei Nie, Xiaokang Lv, Yan Liang, Jinling Hua, Aixin Liang, Liguo Yang

**Affiliations:** 1 College of Animal Science, Anhui Science and Technology University, Chuzhou 233100, China; 2 Key Laboratory of Agricultural Animal Genetics, Breeding and Reproduction of Ministry of Education, College of Animal Science and Technology, Huazhong Agricultural University, Wuhan 430070, China; lax.pipi@mail.hzau.edu.cn; 3 Anhui Province Key Laboratory of Embryo Development and Reproductive Regulation, School of Biological and Food Engineering, Fuyang Normal University, Fuyang 236037, China; 4 Hunan Institute of Animal and Veterinary Science, Changsha 410125, China

**Keywords:** AMH-INH-RFRP, trivalent DNA vaccine, reproduction, dominant follicle, buffalo

## Abstract

Background: Buffaloes have a significant share in the agricultural economies of many developing countries; however, the industry is not well-established because of the low reproductive efficiency of buffaloes. In this study, we designed a novel strategy to enhance buffalo reproductive efficiency and examined the significance of intramuscular AMH-INH-RFRP DNA vaccine immunization on reproductive performance. Methods: Allocated into four groups (*n* = 30/group), female buffaloes (aged 5–8 years, *n* = 120) were randomly enrolled in the study. Treatment groups received 10 mL of AMH-INH-RFRP DNA vaccine intramuscularly, once daily for three consecutive days, at the following concentrations: T1—3 × 10^8^ CFU/mL, T2—3 × 10^9^ CFU/mL, and T3—3 × 10^10^ CFU/mL. All vaccinated groups received a booster vaccination two weeks later, while the control group received 10 mL of phosphate-buffered saline (PBS) intramuscularly on the same schedule. Serum antibody titers against anti-Müllerian hormone (AMH), inhibin (INH), and RF-amide-related peptide (RFRP) were quantified using indirect ELISA on days 14 (post-primary immunization) and 28 (post-booster immunization). Results: Following primary immunization, antibody titers against AMH, INH, and RFRP were significantly higher in the T3 group than in the T1 group (*p* < 0.05), and booster immunization further increased antibody positivity rates. Serum concentrations of IL-4, IFN-γ, and E_2_ were significantly elevated in the T2 and T3 groups compared to the control group (*p* < 0.05), while P_4_ levels showed no significant differences among groups. Ultrasonography revealed that the ovulatory follicle diameter and dominant follicle growth rate were significantly increased in the T2 and T3 groups compared to the control and T1 groups (*p* < 0.05). The estrus rate was significantly higher in the T3 group than in the control group (76.67% vs. 40.00%, *p* < 0.05), and ovulation rates were significantly higher in the T2 (83.33%) and T3 (86.67%) groups compared to the control group (56.67%) (*p* < 0.05). However, conception rates did not differ significantly between vaccinated and control groups in the primary dose comparison (*p* > 0.05). In exploratory post hoc analyses, antibody-positive (Ab+) buffaloes exhibited higher E_2_ concentrations, larger ovulatory follicle diameters, accelerated dominant follicle growth rates, and higher estrus and ovulation rates compared to antibody-negative (Ab−) buffaloes. Furthermore, gestating buffaloes were monitored until calving, revealing no significant effect of the vaccine on newborn calf weight or body size. Conclusions: In total, intramuscular immunization with the AMH-INH-RFRP DNA vaccine, particularly at the highest dose, enhances reproductive performance in buffaloes by stimulating E_2_ secretion and follicular development.

## Simple Summary

Buffaloes are vital for food and farming in many developing countries, but their low reproductive efficiency limits productivity. This study tested an intramuscular DNA vaccine targeting three reproductive hormones in buffaloes. The vaccine, especially at the highest dose, increased immunity, estrogen levels, and follicle growth, leading to significantly higher estrus and ovulation rates. Exploratory analyses further revealed that seropositive buffaloes exhibited improved reproductive parameters, providing associative evidence supporting the vaccine’s mechanism. Conception rates showed numerical improvement, but the increase was not statistically significant. Importantly, it did not affect the neonatal morphometric parameters (birth weight, body length, body height, and chest circumference) of calves. This vaccine offers a practical strategy to enhance buffalo reproduction and support agricultural economies.

## 1. Introduction

Water buffaloes have great significance in ensuring food security and helping transportation in many developing countries, as they provide milk, meat, and draft power. Buffaloes contribute a major share of milk production in India, Pakistan, several Middle Eastern countries, and Italy. In certain regions, crossbreeding programs (Pakistani Nili-Ravi/Indian Murrah buffaloes) have enhanced the economic traits of local dairy buffalo populations [1]. For instance, such crossbreeding has increased the annual milk yield of Chinese buffaloes from 700 kg to 2000 kg [2]. Buffaloes exhibit a strong tolerance to roughage, heat, and harsh environmental conditions. Despite these advantages, their reproductive efficiency remains comparatively low on a global scale. Common reproductive challenges include delayed puberty; weak estrus signs; prolonged postpartum ovarian inactivity; and, most critically, low conception rates, particularly under intensive management systems [3,4]. These reproductive inefficiencies are influenced by multiple factors. Delayed puberty in buffaloes is primarily associated with inadequate nutritional management, seasonal effects on photoperiod and temperature, and genetic factors that affect the onset of cyclicity [3]. Weak estrus signs—a major barrier to timely artificial insemination—are attributed to lower circulating estradiol-17β concentrations compared to cattle, resulting in reduced intensity and duration of behavioral estrus [1,5]. Prolonged postpartum ovarian inactivity is often exacerbated by a negative energy balance during early lactation, suckling intensity, and a poor body condition score, which suppress the hypothalamic-pituitary–ovarian axis function [4,6]. Together, these factors contribute to the low reproductive efficiency that limits buffalo productivity. Reproductive techniques developed from procedures for cattle, when applied to buffaloes, resulted in variable success rates, which include sperm cryopreservation, artificial insemination (AI), and embryonic technologies such as in vitro embryo production and cloning [7]. However, as the potential of buffaloes has been increasingly recognized, research focus on them has intensified. Consequently, the development and application of buffalo-specific reproductive techniques have progressively improved, with methods like AI and in vitro embryo transfer now achieving efficacy comparable to that in cattle [8,9]. Nevertheless, multiple ovulation and embryo transfer (MOET) has demonstrated low embryo recovery rates in buffaloes, limiting its application in this species [7,9,10]. Therefore, our study aims to develop novel strategies to enhance buffalo reproductive efficiency, thereby promoting the healthy and sustainable development of the buffalo industry.

DNA vaccines emerged as a focus of scientific attention in the early 1990s. In 1993, Ulmer et al. [11] established that plasmid DNA induces adaptive immune responses against plasmid-encoded antigens, suggesting its potential as a human vaccine for novel therapeutic applications. These applications include the prevention of various pathogenic infections, as well as the treatment of autoimmunity, allergies, neurological disorders, and cancers [12]. Furthermore, compared to traditional vaccines, the advantages of DNA vaccines, such as their easy design and manufacture, economically cheap cost, convenient transportation, absence of unsafe infectious agents, and broad range of immunogenic epitopes, make them more potent [13]. Consequently, DNA vaccines exhibit considerable potential for application. In veterinary medicine, several vaccines for animal and fish diseases, such as West Nile virus (horse), melanoma (dog), and infectious hematopoietic necrosis virus (salmon), have been approved [14]. Nevertheless, although some studies have explored DNA vaccines for promoting animal reproduction, none have yet been approved for this purpose.

Various DNA vaccines targeting reproductive efficiency in animals have been developed, including those inducing antibody titers against anti-Müllerian hormone (AMH), inhibin (INH), and RF-amide-related peptide (RFRP), and INH-RFRP DNA, demonstrating pro-reproductive effects across species. Previous studies have reported an increase in large follicle counts and litter size in rats and sheep when immunized with an INH DNA vaccine [15,16]. Further, varying concentrations of the INH DNA vaccine administered via intramuscular injection promote follicular development and growth in buffaloes, thereby improving estrus performance, ovulation rates, and, ultimately, conception rates [17]. Yu et al. [18] immunized mice with INH and AMH DNA vaccines, revealing that both vaccines enhanced estrogen secretion and significantly increased litter size, with combined INH+AMH immunization yielding superior reproductive outcomes compared to individual vaccines. Dan et al. [19] developed an INH-RFRP DNA vaccine that significantly increased litter size in mice. Subsequently, the same vaccine was tested in Tan sheep [20]; intramuscular immunization successfully induced humoral immunity and elevated twin-lambing rates in ewes. Collectively, these findings demonstrate that DNA vaccine immunization enhances reproductive efficiency in animals and that combinatorial approaches are more effective than single-antigen vaccines. Based on this evidence, our laboratory explored an AMH-INH-RFRP DNA vaccine. Therefore, the present study aimed to estimate whether intramuscular immunization with the trivalent AMH-INH-RFRP DNA vaccine impacts reproductive parameters, including estrus, ovulation, and conception rates in buffaloes, as well as the subsequent growth of their calves.

## 2. Materials and Methods

### 2.1. DNA Vaccine Preparation

The AMH-INH-RFRP DNA vaccine was produced by our laboratory. For specific details, please refer to our previous description in [21]. For vaccine preparation, an inoculum (200 mL LB liquid medium obtained from Beijing Solarbio Science & Technology Co., Ltd., Beijing, China) containing the DNA vaccine (1:100 dilution) was cultured (37 °C; 16 h) with continuous shaking (220 rpm). Bacterial cells were then pelleted by centrifugation (4 °C, 10 min) and re-suspended in PBS. Prior to immunization, the bacterial suspension was serially diluted and spread-plated in triplicate onto LB agar plates. After incubation at 37 °C for 16–18 h, colonies were counted, and the colony-forming units (CFU) per milliliter were calculated to determine the final vaccine concentration. Phosphate-buffered saline (PBS; Beijing Solarbio Science & Technology Co., Ltd., Beijing, China) was used to adjust the final concentration to 3 × 10^10^ CFU/mL.

### 2.2. Animals and Immunization Procedure

Healthy multiparous buffaloes were sourced from a buffalo farm (Hubei Jinniu Animal Husbandry Co., Ltd., Shayang, Jingmen, China). All animals received a total mixed ration of forage and concentrate with ad libitum water access. Animal use protocols were approved by the Experimental Animal Management and Ethics Committee of Huazhong Agricultural University (HZAUBU-2019-001).

**Figure 1:**
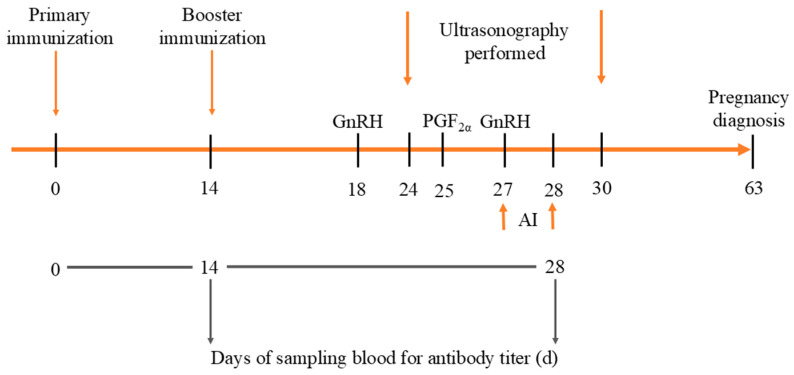
DNA vaccine immunization flowchart. Buffaloes in the experimental groups received the AMH-INH-RFRP DNA vaccine via intramuscular injection (IM) on days 0 and 14, whereas PBS was injected into the control group. At day 4 post-booster immunization, all buffaloes were subjected to the Ovsynch-TAI procedure. Ultrasonography was performed every 12 h from days 24 to 30 to assess follicular development and ovulation. On day 63, all buffaloes were examined for pregnancy diagnosis.

A total of 120 postpartum buffaloes were randomly allocated to four groups (*n* = 30 per group). Randomization was employed to ensure a balanced distribution of baseline characteristics (e.g., age, parity, and body condition) across groups. Treatment groups received intramuscular injections of 10 mL AMH-INH-RFRP DNA vaccine once daily for three consecutive days at the following concentrations: T1—3 × 10^8^ CFU/mL, T2—3 × 10^9^ CFU/mL, and T3—3 × 10^10^ CFU/mL. The control group received 10 mL PBS intramuscularly on the same schedule. Two weeks post-primary immunization, all groups received booster vaccinations following identical protocols. Concurrently, buffaloes underwent the classical Ovsynch-timed artificial insemination protocol (Ovsynch-TAI) protocol for estrus synchronization. Blood samples collected on days 14 and 28 post-immunization from the jugular vein were centrifuged (3000 rpm, 10 min, 4 °C) to separate the serum. The collected serum was stored at −80° until analysis. Experimental procedures are summarized in Figure 1.

### 2.3. Estrus Synchronization Procedure and Ultrasonographic Detection

The AMH-INH-RFRP DNA vaccine was administered in conjunction with an Ovsynch-TAI protocol. The Ovsynch-TAI regimen comprised the following steps: administration of gonadotropin-releasing hormone (GnRH) (200 μg, i.m.) on day 18, administration of prostaglandin F_2_α (PGF_2_α; 0.4 mg, i.m.) on day 25, and a second administration of GnRH (200 μg, i.m.) on day 27. All buffaloes underwent artificial insemination 18–24 h after the second GnRH injection. The hormones used in this protocol were obtained from Ningbo Sansheng Biological Technology Co., Ltd. (Ningbo, China).

From day 24 (24 h pre-PGF_2_α) to day 30 (72 h post-second GnRH), transrectal ultrasonography (Shenzhen Well. D Medical Electronics Co., Ltd., Shenzhen, China) was performed at 12 h intervals to monitor follicular dynamics and ovulation. The ultrasonographer was blinded to both the treatment group allocation (Ctrl/T1/T2/T3) and serological status (Ab+/Ab−) throughout all measurements to minimize observer bias. Estrus was confirmed twice daily (08:00 and 16:00) through the observation of copious vaginal mucus. Ovulation was defined as the disappearance of the dominant follicle (≥10 mm in diameter) between two consecutive scans.

Follicular growth rate (mm/day) was calculated as the difference in diameter of the dominant follicle between two consecutive daily measurements, divided by the time interval (24 h). Specifically, the growth rate for each animal was derived from the linear slope of follicle diameter measurements recorded daily from day 24 to day 30, and the values were expressed as mean ± SEM per group. Pregnancy diagnosis via ultrasonography was conducted 35 days post-AI to determine conception rates.

### 2.4. Detection of Antibody Titer

Synthetic antigens (INH, AMH, and RFRP) from Sangon Biotech (Ningbo, China) were used in indirect ELISA to quantify antibodies. For specific details, please refer to our previous report in [21]. The positivity threshold (cut-off value) was defined as the mean OD_450_ of the negative control (PBS-immunized control sera) plus two standard deviations (mean + 2SD), calculated independently for each assay plate. Samples with OD_450_ values exceeding the threshold at a given dilution were considered positive, and their antibody titers were expressed as the reciprocal of the highest dilution that yielded a positive result. Samples with OD_450_ values ≤ (mean + 2SD) were scored as negative. Based on the antibody titers measured after booster immunization, buffaloes were classified as seropositive (Ab+) if they exhibited positive responses against at least two of the three target antigens (AMH, INH, and RFRP); animals that failed to meet this criterion (positive against only one antigen or none) were classified as seronegative (Ab−). This definition was applied uniformly across all treatment and control groups for exploratory post hoc subgroup analyses. The ELISA assay was validated using pre-immune serum samples as paired negative controls to confirm specificity. Intra- and inter-assay coefficients of variation were <10% and <15%, respectively, for all three antigens. Control group data were used exclusively for threshold determination. These criteria were established according to previously published methods [19].

### 2.5. Assay of Reproductive Hormone and Cytokine

Serum concentrations of interleukin-4 (IL-4), interferon-gamma (IFN-γ), estradiol (E_2_), and progesterone (P_4_) were determined using commercial ELISA kits (bovine, Ruixin Biological Technology Co., Ltd., Quanzhou, China; Cat# RX1600854B, RX1600863B, RXJ1600845B, and RXJ1600748B), in accordance with the manufacturers’ instructions. Blood samples for these assays were collected on day 28 post-immunization. These commercial ELISA kits, although developed for bovine samples, have been widely used in previous buffalo studies [17,22], providing empirical support for their applicability in this species. Assay validation parameters showed that intra- and inter-assay coefficients of variation were less than 10% and 15% for IL-4, and less than 15% for in the IFN-γ, E_2_, and P_4_ assays.

### 2.6. Morphometric Measurements of Newborn Calves

Within 24 h after birth, all calves were weighed using a digital livestock scale (Shanghai Yaohua Weighing System Co., Ltd., Shanghai, China) to the nearest 0.01 kg. Body length was measured as the straight distance from the point of the shoulder (tuberosity of the humerus) to the pin bone (tuber ischii) using a standard measuring tape. Body height was measured as the vertical distance from the ground to the highest point of the withers (spinous process of the sixth thoracic vertebra). Chest circumference (heart girth) was measured as the perimeter around the chest immediately caudal to the forelegs, at the level of the olecranon. All measurements were performed by the same trained technician to minimize inter-observer variability.

### 2.7. Statistical Analyses

The randomized dose-group comparisons (Ctrl, T1, T2, T3) for antibody titers, hormone concentrations, follicular development, and reproductive rates were designated as the primary analysis to support causal inference. Differences among these groups were assessed using one-way ANOVA followed by Tukey’s post hoc test, and chi-square tests were used for rates. All statistical analyses were performed using SPSS software (version 27.0, IBM Corp., Armonk, NY, USA).

The stratification of samples based on post-immunization antibody status (Ab+ vs. Ab−) was defined as an exploratory post hoc subgroup analysis. These comparisons were performed using independent *t*-tests for continuous variables and chi-square tests for categorical variables, and the results are presented to explore associations between serological responsiveness and reproductive parameters rather than to infer direct causation. A *p* < 0.05 was considered statistically significant, while *p* < 0.01 was set to indicate high significance.

## 3. Results

### 3.1. Immune Response in Buffaloes Immunized with the AMH-INH-RFRP DNA Vaccine

**Figure 2:**
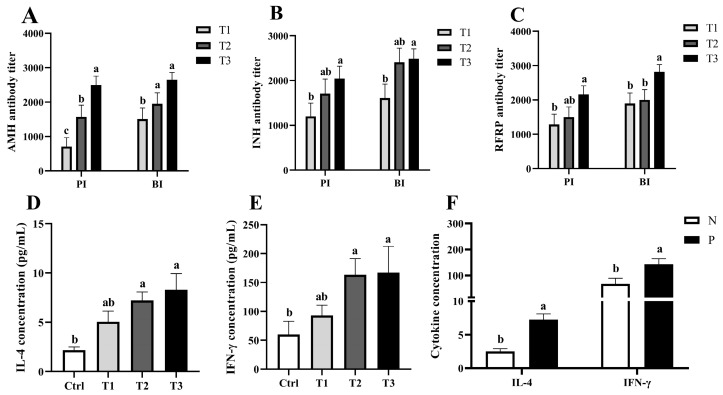
Immune response in buffaloes immunized with different concentrations of the AMH-INH-RFRP DNA vaccine. Antibody titers against AMH (**A**), INH (**B**), and RFRP (**C**), as well as serum IL-4 (**D**) and IFN-γ (**E**) concentrations, were detected by ELISA; (**F**) serum IL-4 and IFN-γ concentrations in Ab+ and Ab− buffaloes were also determined (via exploratory post hoc analysis). PI: primary immunization; BI: booster immunization; P: Ab+; N: Ab−. Data are presented as means ± SEM. Bars with different letters indicate significant differences (*p* < 0.05).

After the initial immunization, the T2 and T3 groups showed significantly higher levels of antibody (anti-AMH) titers than the T1 group (*p* < 0.05), whereas the antibody (anti-AMH and anti-RFRP) titers were significantly higher in the T3 group than in the T1 group (*p* < 0.05) (Figure 2A). Post-booster immunization, the T2 and T3 groups showed significantly higher levels of anti-AMH antibody titers than the T1 group (*p* < 0.05), the anti-INH antibody titers in the T3 group were significantly higher than in the T1 group (*p* < 0.01), and the anti-RFRP antibody titers in the T3 group were significantly higher than in the T1 and T2 groups (*p* < 0.05) (Figure 2A–C). In addition, the levels of antibody (anti-AMH, anti-INH, and anti-RFRP) titers after the booster immunization were higher than those after the initial immunization (Figure 2A–C).

Serum concentrations of Th1/Th2-type cytokines (IL-4, IFN-γ) were quantified using ELISA. The results showed that buffaloes in the T2 and T3 groups had significantly higher serum levels of IL-4 and IFN-γ than those in the control group (*p* < 0.05) (Figure 2D,E). Furthermore, in the exploratory post hoc analyses, Ab+ buffaloes exhibited markedly higher IL-4 and IFN-γ concentrations compared to Ab− buffaloes (*p* < 0.05) (Figure 2F), indicating an association between seropositivity and enhanced immune activation.

Antibody positivity (AMH, INH, and RFRP) rates across the DNA vaccine concentrations were comparatively analyzed (Table 1). Following the primary immunization, the positive rates of anti-AMH and anti-INH antibodies were significantly higher in the T2 and T3 groups than in the T1 group (*p* < 0.05). Additionally, the T3 group demonstrated a 30.00% absolute increase in antibody positivity (anti-RFRP) compared to the T1 group (*p* < 0.05). Post-booster immunization, all antibody positivity rates exceeded 90.00%. Despite no significant differences found among the treatment groups after the booster immunization, the booster dose increased the antibody positivity rates compared to those after the primary immunization.

### 3.2. Follicular Development in Buffaloes Immunized with the AMH-INH-RFRP DNA Vaccine

As shown in Table 2, the diameter of ovulatory follicles was significantly larger (*p* < 0.05) in the T2 (12.70 ± 0.18 mm) and T3 (13.52 ± 0.21 mm) groups compared to the control (11.30 ± 0.26 mm) and T1 (11.80 ± 0.18 mm) groups. In addition, the growth rate of the dominant follicle was significantly higher (*p* < 0.05) in the T2 (1.56 ± 0.04 mm/day) and T3 (1.64 ± 0.03 mm/day) groups compared to the control (1.23 ± 0.04 mm/day) and T1 (1.28 ± 0.06 mm/day) groups.

**Figure 3:**
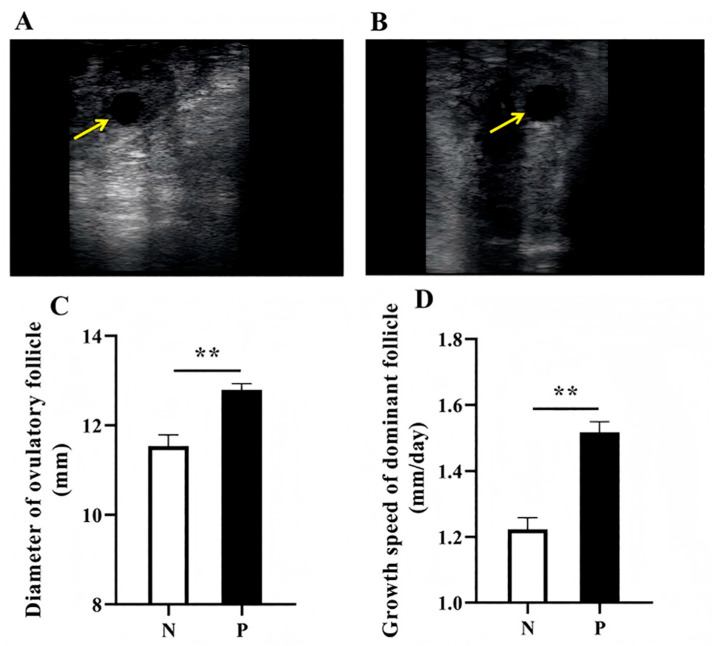
Exploratory post hoc analysis of follicular development in Ab+ and Ab− buffaloes. (**A**,**B**) B-type ultrasound photographs of dominant follicles in antibody-negative (Ab− = N) and antibody-positive (Ab+ = P) buffaloes, respectively (yellow arrows refer to the dominant follicle); (**C**) diameter of ovulatory follicles in Ab+ and Ab− buffaloes; (**D**) growth speed of dominant follicles in Ab+ and Ab− buffaloes. Data are presented as means ± SEM. * Significant difference (*p* < 0.05), ** highly significant differences (*p* < 0.01).

Comparative analysis of follicular dynamics between Ab+ and Ab− buffaloes was performed as an exploratory post hoc analysis (Figure 3). Results showed a significant increase of 1.25 mm in ovulatory follicle diameter from the Ab+ group compared to the Ab− group (*p* < 0.01) (Figure 3A–C), suggesting an association between seropositivity and enhanced follicular maturation. Concurrently, the growth rate of dominant follicle was markedly accelerated in the Ab+ group (1.52 ± 0.24 mm/day) versus the Ab− group (1.22 ± 0.17 mm/day) (*p* < 0.01) (Figure 3D).

### 3.3. Reproductive Hormones in Buffaloes Immunized with the AMH-INH-RFRP DNA Vaccine

**Figure 4:**
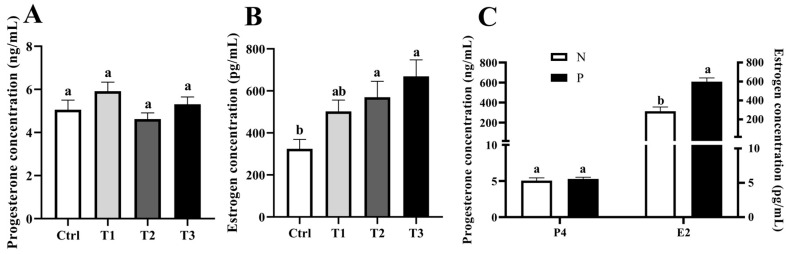
Concentrations of reproductive hormones in buffaloes. (**A**,**B**) Effects of the DNA vaccine on P_4_ and E_2_, respectively; (**C**) concentrations of P_4_ and E_2_ in Ab+ and Ab− buffaloes (exploratory post hoc analysis). Data are presented as means ± SEM. Bars with different letters indicate significant differences (*p* < 0.05).

Serum P_4_ and E_2_ concentrations are presented in Figure 4A–C. P_4_ levels showed no significant differences among all treatment groups (*p* > 0.05). In contrast, the E_2_ concentrations in the T2 and T3 groups significantly increased by 245.29 pg/mL and 344.72 pg/mL compared to the control group (*p* < 0.05). In exploratory post hoc analyses, Ab+ buffaloes exhibited significantly higher E_2_ levels compared to Ab− buffaloes (*p* < 0.05), whereas P_4_ concentrations exhibited no intergroup differences (*p* > 0.05).

### 3.4. Estrus, Ovulation, and Conception Rates in Buffaloes Immunized with the AMH-INH-RFRP DNA Vaccine

Reproductive performance data are presented in Table 3. The estrus rate was significantly higher in the T3 group than in the control group (76.67% vs. 40.00%, *p* < 0.05). Ovulation rates were significantly higher in the T2 (83.33%) and T3 (86.67%) groups compared to the control group (56.67%) (*p* < 0.05). Conception rates showed numerical increases in the T2 (40.00%) and T3 (46.67%) groups compared to the control group (23.33%), but these differences were not statistically significant (*p* > 0.05).

To clarify the allocation of animals across serological status groups, a contingency table of treatment group × antibody status (Ab+/Ab−) was constructed based on the criterion of positivity against at least two of the three target antigens after booster immunization (Table 4). As shown, all 30 control animals were classified as Ab− due to the absence of specific antibody responses. Among vaccinated animals, 85 out of 90 exhibited seropositivity (≥2 antigen-positive), while only five vaccinated animals across all treatment groups (*n* = 3 in T1, *n* = 1 in T2, *n* = 1 in T3) failed to mount a detectable antibody response against at least two antigens and were thus classified as Ab−.

In exploratory post hoc analyses (Table 5), Ab+ buffaloes showed significantly elevated estrus and ovulation rates compared to Ab− buffaloes (*p* < 0.01). Ab+ buffaloes exhibited a 24.04% higher conception rate than Ab− buffaloes (*p* < 0.05). Collectively, the primary randomized comparison (Table 3) demonstrates significant improvements in estrus and ovulation rates in vaccinated groups, while exploratory post hoc analyses (Table 5) show associative evidence linking seropositivity with enhanced reproductive performance.

### 3.5. Morphometric Parameters of Buffalo Calves

Neonatal morphometric parameters are presented in Table 6. The vaccinated and control groups showed no significant differences (*p* > 0.05) regarding birth weight, body height, body length, and chest circumference. These findings demonstrate that maternal vaccination had no adverse effects on neonatal morphometric measurements.

## 4. Discussion

DNA vaccines offer several advantages over traditional vaccines, including ease of design and manufacture, cost-effectiveness, and the ability to induce broad immune responses. However, their limited immunogenicity remains a major challenge for practical application [13]. In the present study, intramuscular administration of the trivalent AMH-INH-RFRP DNA vaccine at the highest dose (T3: 3 × 10^10^ CFU/mL) induced antibody positivity rates exceeding 90% against all three target antigens (AMH, INH, and RFRP) after booster immunization, with the T3 group showing significantly higher antibody titers compared to the T1 group. Furthermore, antibody-positive (Ab+) buffaloes exhibited elevated levels of both IFN-γ (a Th1 cytokine associated with cellular immunity) [23] and IL-4 (a Th2 cytokine associated with humoral immunity) [24], suggesting that the vaccine elicited a balanced Th1/Th2 immune response. We attribute the robust immunogenicity observed in this study to two key design features. First, the inclusion of the CMV promoter in the plasmid backbone likely enhanced transgene expression [22,25], while the fusion of each target gene with the hepatitis B surface antigen gene (HBsAg-S) may have facilitated antigen presentation and improved the recruitment of immune cells to the site of immunization [26]. Second, the relatively high vaccination dose (3 × 10^10^ CFU/mL) and the intramuscular route of administration—which ensures efficient antigen uptake by antigen-presenting cells—likely contributed to the superior humoral and cellular responses observed in the T3 group. Our findings are consistent with previous reports demonstrating the immunogenicity of INH DNA vaccines in cattle and buffaloes. Meng et al. [27] reported that an INH DNA vaccine delivered intramuscularly to beef cattle achieved an antibody positivity rate exceeding 90% only at the highest dose (1.5 mg). Similarly, Liu et al. [17] found that in buffaloes, the high-dose group (1 × 10^10^ CFU/mL) reached an antibody positivity rate of 90.2% after booster immunization, while the medium- and low-dose groups achieved lower rates. In contrast to these single-antigen vaccines, our trivalent vaccine induced positivity rates exceeding 90% against all three target antigens simultaneously, indicating that the HBsAg-S fusion strategy effectively overcame the immunogenicity limitations typically associated with DNA vaccines.

Interestingly, these results contrast with our previous study using the same AMH-INH-RFRP DNA vaccine delivered via the nasal route in buffaloes, where the high-dose group (3 × 10^10^ CFU/mL) did not achieve the same level of antibody response as the medium-dose group [21]. The superior performance of intramuscular delivery over nasal delivery in the present study may be attributed to more efficient antigen uptake and presentation at the injection site, as well as the avoidance of physical barriers (e.g., nasal mucosal clearance and sneezing) that likely reduced vaccine retention and antigen exposure in the nasal immunization study [21]. Notably, the intramuscular protocol also reduced the overall immunization timeline by 10 days compared to the nasal protocol, while achieving superior outcomes in estrus and ovulation rates, thereby providing enhanced practical feasibility and economic efficiency for buffalo farming operations.

Subestrus or silent estrus is probably the most important factor contributing to low reproductive efficiency in buffaloes [28], and proper estrus detection is critical for artificial insemination (AI) practices to maximize reproductive efficiency [29]. Studies on estrous behavior and endocrinology in buffaloes indicate considerable variation in reproductive endocrine activity, and the absence of external signs of estrus is common [5,6]. The lower intensity of estrus in buffaloes compared to cows may be due to lower circulating concentrations of estradiol-17β (E_2_) [1]. An increase in the frequency and duration of estrogen secretion contributes not only to the intensity of behavioral estrus, but also to the duration of estrus [30].

The current study found that intramuscular immunization with the AMH-INH-RFRP DNA vaccine significantly increased serum circulating estrogen concentrations and improved estrus rates. The primary randomized comparison demonstrated a 36.67% increase in the estrus rate in the T3 group compared to the control group. Furthermore, estrogen plays a fundamental role in regulating the endocrine and behavioral events connected with the estrous cycle. E_2_ induces a surge of luteinizing hormone (LH) prior to ovulation as an “all or nothing” event. Upon reaching a certain threshold, E_2_ sends a signal for the occurrence of the LH surge, which culminates in ovulation [31]. In the present study, the primary dose-comparison data showed that ovulation rates in the T2 and T3 groups were significantly higher than in the control group by 26.66% and 30.00%, respectively, confirming the causal efficacy of the vaccine. Furthermore, the exploratory post hoc analysis revealed that Ab+ buffaloes had significantly higher estrus and ovulation rates compared to Ab− buffaloes, which is consistent with the primary findings showing a threshold effect at higher doses. Although these associative results should be interpreted with caution due to the potential for selection bias (e.g., inherent differences in health or immune competence among responders), they provide correlative support for the hypothesis that the vaccine enhances reproductive performance through the neutralization of AMH, INH, and RFRP, leading to elevated estrogen levels and improved follicular development.

AMH plays a crucial role in ovarian function, and it was found that the level of AMH gene expression in granulosa cells (GCs) of medium follicles was significantly higher than that of large follicles [32]; moreover, the concentration of AMH in follicular fluid declined with increasing follicular diameter, and exhibited a positive correlation with the number of antral follicles [33]. Meanwhile, regulation of ovarian function through negative feedback mediated by the inhibitory effects of INH on FSH via the pituitary–gonadal axis has been reported [34], while RFRP inhibits follicular development and estrogen secretion by regulating the function of ovarian granulosa cells in the gonads [35,36]. In our study, we found that the diameter of ovulatory follicles and the growth rate of dominant follicles were significantly higher in the Ab+ group than in the Ab− group, which may be attributed to active immunization with the AMH-INH-RFRP DNA vaccine, which neutralized the biological activity of AMH, INH, and RFRP levels, thereby promoting follicular development and E_2_ secretion. However, it is important to acknowledge the compositional nature of the Ab− group in this exploratory analysis. As shown in Table 4, the Ab− group comprises all 30 control animals, plus the only five vaccinated animals (3 in T1, 1 in T2, and 1 in T3) that failed to develop a positive antibody response against at least two of the three target antigens. Thus, the Ab+ versus Ab− comparison overwhelmingly represents a contrast between vaccinated responders (*n* = 85) and unvaccinated controls (*n* = 30), with vaccinated non-responders contributing only marginally (5/35, 14.29%) to the Ab− group. While this overlap may introduce potential confounding factors—such as inherent differences in health status, metabolic condition, or baseline immune competence between responders and non-responders—the small proportion of vaccinated non-responders within the Ab− group limits the practical impact of this limitation on the overall interpretation. Nevertheless, these exploratory findings should be interpreted with caution and are presented primarily as associative evidence consistent with the primary finding of a threshold effect at higher doses. The causal efficacy of the vaccine is established by the randomized dose-comparison data (T3 vs. Ctrl), which form the primary basis for our conclusions.

In this study, all buffaloes, regardless of treatment group, received the same Ovsynch-TAI protocol for estrus synchronization. This standardized hormonal regimen was applied uniformly across all groups, ensuring that any observed differences in estrus, ovulation, and conception rates between vaccinated and control animals are attributable to the vaccine rather than to the synchronization procedure itself. The Ovsynch-TAI protocol effectively synchronizes follicular wave emergence and ovulation through precisely timed administration of GnRH and PGF_2_α, allowing for timed artificial insemination without the need for estrus detection [9]. In our study, the combination of the AMH-INH-RFRP DNA vaccination with Ovsynch-TAI achieved estrus rates of up to 76.67% and ovulation rates of up to 86.67% in the T3 group, compared to 40.00% and 56.67% in the control group, respectively. These findings suggest that the vaccine synergizes with the synchronization protocol by promoting follicular development and enhancing estrogen secretion, thereby improving the response to the hormonal regimen. While Ovsynch-TAI alone improves reproductive management through synchronization, the addition of the DNA vaccine provides an immunological strategy to further enhance ovarian function and fertility outcomes.

On the other hand, the birth weight and body size of calves are important quantitative traits and key factors affecting lactation and reproduction of cattle, which are closely related to production efficiency [37]. Newborn calf weight and body size indirectly correlates with calf morbidity, while directly correlating not only with calf survival at calving, but also the promotion of fast growth and longevity [38,39]. Furthermore, the birth weight of calves can reflect early embryonic development and later growth of calves, and, to some extent, affects the weight and productivity of calves in adulthood [40]. Therefore, the present experiment evaluated whether AMH-INH-RFRP DNA vaccine immunization influenced birth weight and other growth parameters of calves born to pregnant female buffaloes. Our results showed that the vaccine had no significant effects on calf body measurements (birth weight, body length, body height, and chest circumference).

Several limitations of this study should be acknowledged. First, while the ultrasonographer was blinded to group assignments, subjective variation in follicular boundary identification may still have contributed to minor measurement variability. Second, baseline parameters that may influence reproductive performance—such as age, parity, days postpartum, and body condition score—were not systematically recorded prior to treatment initiation. Although random allocation of the 120 buffaloes across the four groups should have ensured comparable distribution of these characteristics, the absence of these data prevents formal statistical confirmation. Future studies should systematically record and report these parameters to enhance transparency. Third, although the synthetic peptides used as coating antigens for AMH, INH, and RFRP share no significant sequence homology based on alignment analysis—suggesting minimal risk of cross-reactivity among the three targets—direct cross-reactivity testing was not performed. Fourth, the commercial ELISA kits used for cytokine and hormone detection were developed for bovine samples; although these kits have been widely used in previous buffalo research [17,22], we did not conduct species-specific validation in this study. Fifth, as discussed above, the exploratory Ab+/Ab− subgroup analyses are associative rather than causal. Despite these limitations, the primary randomized dose-comparison data provide robust evidence for the vaccine’s efficacy.

## 5. Conclusions

In conclusion, our randomized controlled data demonstrate that intramuscular administration of the AMH-INH-RFRP DNA vaccine, particularly at the highest dose (T3), causally enhances estrus and ovulation rates in buffaloes by neutralizing target hormones of AMH, INH, and RFRP, thereby increasing systemic estrogen levels and promoting follicular development. However, conception rates did not differ significantly between the vaccinated and control groups in the primary dose comparison. The exploratory Ab+/Ab− comparisons are hypothesis-generating and suggest associations between seropositivity and improved reproductive parameters that warrant further investigation; they should not be interpreted as evidence of vaccine efficacy. Crucially, maternal vaccination exerted no adverse effects on neonatal morphometric measurements at birth. Collectively, integrating AMH-INH-RFRP immunization with the Ovsynch-TAI protocol enhances estrus and ovulation rates in buffalo herds, while the impact on conception rates remains to be established.

## Figures and Tables

**Table 1:** Percentage of antibody-positive buffaloes (%) against AMH, RFRP, and INH at various intervals following immunization with different concentrations of the vaccine.

Group	Number	Primary Immunization			Booster Immunization		
		Anti-AMH	Anti-INH	Anti-RFRP	Anti-AMH	Anti-INH	Anti-RFRP
T1	30	66.67 ^b^ (20/30)	70.00 ^b^ (21/30)	66.67 ^b^ (20/30)	93.33 (28/30)	93.33 (28/30)	90.00 (27/30)
T2	30	96.67 ^a^ (29/30)	93.33 ^a^ (28/30)	80.00 ^ab^ (24/30)	100.00 (30/30)	96.67 (29/30)	90.00 (27/30)
T3	30	96.67 ^a^ (29/30)	93.33 ^a^ (28/30)	96.67 ^a^ (29/30)	100.00 (30/30)	96.67 (29/30)	96.67 (29/30)

Within the same column, values with different lowercase superscript letters indicate significant differences (*p* < 0.05), whereas values with no letter or the same lowercase superscript letter indicate no significant differences (*p* > 0.05). These values represent positivity rates for each individual antigen, which served as the basis for the composite Ab+/Ab− classification defined in Section 2.3.

**Table 2:** The ovulatory follicles (diameter) and dominant follicles (growth speed) in buffaloes after immunization with different concentrations of the vaccine.

Groups	Diameter of Ovulatory Follicle (mm)	Growth Speed of Dominant Follicle (mm/day)
Ctrl	11.30 ± 0.26 ^d^	1.23 ± 0.04 ^b^
T1	11.80 ± 0.18 ^cd^	1.28 ± 0.06 ^b^
T2	12.70 ± 0.18 ^b^	1.56 ± 0.04 ^a^
T3	13.52 ± 0.21 ^a^	1.64 ± 0.03 ^a^

Within the same column, values with different lowercase superscript letters indicate significant differences (*p* < 0.05), whereas values with no letter or the same lowercase superscript letter indicate no significant differences (*p* > 0.05). Data are expressed as means ± SEM.

**Table 3:** Rates of estrus, ovulation, and conception among different groups (primary randomized comparison).

Variable	Treatment Groups			
	Ctrl	T1	T2	T3
Estrus (%)	40.00 ^b^ (12/30)	56.67 ^ab^ (17/30)	60.00 ^ab^ (18/30)	76.67 ^a^ (23/30)
Ovulation (%)	56.67 ^b^ (17/30)	80.00 ^ab^ (24/30)	83.33 ^a^ (25/30)	86.67 ^a^ (26/30)
Conception (%)	23.33 (7/30)	26.67 (8/30)	40.00 (12/30)	46.67 (14/30)

Data are presented as percentages with absolute numbers in parentheses. Within the same row, values with different lowercase superscript letters indicate significant differences (*p* < 0.05), whereas values with no letter or the same lowercase superscript letter indicate no significant difference (*p* > 0.05).

**Table 4:** Contingency table of treatment group × serological status (Ab+/Ab−) after booster immunization.

Group	Total (*n*)	Ab+ (*n*, %)	Ab− (*n*, %)
Ctrl	30	0 (0.00%)	30 (100.00%)
T1	30	27 (90.00%)	3 (10.00%)
T2	30	29 (96.67%)	1 (3.33%)
T3	30	29 (96.67%)	1 (3.33%)
Total	120	85 (70.83%)	35 (29.17%)

**Table 5:** Exploratory post hoc comparison of estrus, ovulation, and conception rates between Ab+ and Ab− buffaloes.

Variable	Ab− (N = 35)	Ab+ (P = 85)
Estrus (%)	40.00 ^b^ (14/35)	65.88 ^a^ (56/85)
Ovulation (%)	54.29 ^b^ (19/35)	85.88 ^a^ (73/85)
Conception (%)	17.14 ^b^ (6/35)	41.18 ^a^ (35/85)

Data are presented as percentages with absolute numbers in parentheses. Within the same row, values with different lowercase superscript letters indicate significant differences (*p* < 0.05), whereas values with no letter or the same lowercase superscript letter indicate no significant difference (*p* > 0.05).

**Table 6:** Effect of DNA vaccine on calf growth.

Group	Newborn Weight (kg)	Body Length (cm)	Body Height (cm)	Chest Circumference (cm)
Ctrl	39.45 ± 4.90	59.14 ± 3.09	70.13 ± 5.07	75.86 ± 4.26
T1	39.27 ± 6.60	59.00 ± 4.70	72.37 ± 6.36	77.15 ± 4.90
T2	38.30 ± 5.92	59.45 ± 2.73	69.42 ± 4.07	74.57 ± 1.45
T3	38.53 ± 5.01	59.43 ± 2.97	71.19 ± 3.53	77.23 ± 4.18

## Data Availability

The data generated and analyzed during the current study are available from the corresponding author upon reasonable request.
